# Guillain-Barré Syndrome following weight loss: a review of five diet-induced cases and nineteen bariatric surgery cases

**DOI:** 10.3389/fneur.2025.1557515

**Published:** 2025-03-25

**Authors:** Qiong Wu, Fang-Yi Li, Jue Hu, Wei Xu, Tie-Qiao Feng, Hua-Shan Zhou, Zhen Wang, Wen-Gao Zeng

**Affiliations:** ^1^Department of Neurosurgery, The Affiliated Changsha Central Hospital, Hengyang Medical School, University of South China, Changsha, China; ^2^Department of Neurology, The Affiliated Changsha Central Hospital, Hengyang Medical School, University of South China, Changsha, China; ^3^Department of Pathology, Changsha Hospital for Maternal and Child Health Care, Affiliated to Hunan Normal University, Changsha, China

**Keywords:** Guillain-Barré Syndrome, weight loss, bariatric surgery, restrictive diets, nutritional deficiencies

## Abstract

**Introduction:**

Obesity is a worldwide health concern frequently addressed by weight reduction strategies, including bariatric surgery and restricted diets. While effective, these approaches can result in complications, including Guillain-Barré Syndrome (GBS), a rare but serious autoimmune disorder. This study aims to analyze clinical and neurophysiological features of diet-induced GBS and compare them to cases linked with bariatric surgery.

**Methods:**

We retrospectively reviewed medical records of five patients admitted to our institution between August 2012 and August 2022, who developed GBS during active dieting resulting in significant weight loss. Clinical presentations, laboratory results, neurophysiological findings, and nutritional status during treatment were analyzed. Additionally, we performed a literature review comparing these cases with nineteen previously reported instances of bariatric surgery-associated GBS.

**Results:**

All five patients exhibited acute, symmetrical limb weakness primarily affecting the lower extremities, accompanied by diminished tendon reflexes. Neurophysiological assessments revealed axonal damage in all cases, and albuminocytologic dissociation was present in two patients. Three patients received intravenous immunoglobulin (IVIG) therapy, while the remaining two underwent nutritional therapy alone. All patients achieved full recovery within 6 months. Notably, the rate of weight loss observed significantly exceeded recommended safe guidelines.

**Discussion:**

Rapid and substantial weight loss may play a role in triggering GBS, possibly due to nutritional deficiencies or immune dysregulation. Clinicians should recognize the potential neurological risks associated with aggressive weight-loss strategies. Early diagnosis and appropriate intervention are crucial for favorable outcomes and preventing complications.

## 1 Introduction

Obesity is a complex and multifactorial condition that has become a global health epidemic, with far-reaching consequences for both individual health and public health systems (1, 2). It is associated with numerous comorbidities, including cardiovascular diseases, type 2 diabetes, and various metabolic disorders (3–6). The growing prevalence of obesity has led to an increasing burden on healthcare systems worldwide (7). As a result, effective interventions for managing obesity are crucial to reducing associated risks and improving the quality of life for affected individuals (8).

Bariatric surgery has emerged as an effective treatment for severe obesity, leading to substantial weight loss and improvement in comorbid conditions (9–11). However, despite its efficacy, bariatric surgery is not without risks. The incidence of peripheral neuropathies, including Guillain-Barré Syndrome (GBS), following weight loss surgery is approximately 0.06% (12–16). In contrast, diet-induced weight loss is a non-invasive alternative that is widely employed to manage obesity. While it is considered safer than surgical interventions, the potential for diet-induced complications, including GBS, remains underexplored.

GBS is an autoimmune disorder characterized by progressive muscle weakness and paralysis, typically following a triggering event such as an infection, trauma, or surgery (17, 18). Although rare, GBS has been reported in patients undergoing significant weight loss through bariatric surgery (15, 19–22). However, instances of GBS associated with dieting remain exceedingly rare, warranting further investigation.

In this study, we report five cases of GBS that developed during active dieting, providing a detailed analysis of these cases and exploring the potential link between weight loss through dieting and the onset of GBS. Additionally, we compare these cases with nineteen previously reported bariatric surgery-associated GBS cases to elucidate potential differences in clinical features, pathophysiological mechanisms, and outcomes.

## 2 Materials and methods

This retrospective study included patients diagnosed with GBS who were actively dieting at the time of symptom onset. We reviewed the medical records of five such patients between August 2012 and August 2022. These patients met the diagnostic criteria for GBS, as outlined by Shahrizaila et al. (23). We collected data on their clinical presentation, laboratory findings, and treatment outcomes. Additionally, a comprehensive literature search was performed using PubMed and Google Scholar databases up to January 2025 to identify similar cases reported in the field. The search included terms such as “weight loss,” “Guillain-Barré Syndrome,” “bariatric surgery” and specific types of bariatric procedures. This study was a retrospective analysis of existing patient records without any particular intervention. Nutritional status, including micronutrient levels, was monitored as part of the clinical management of the patients to identify and address any deficiencies. We ensured full protection of patient privacy and adhered to the Helsinki Declaration. Ethical approval was not required for this retrospective study, as it was based on anonymized medical records.

## 3 Results

### 3.1 Clinical characteristics of diet-induced GBS cases

Table 1 summarizes the clinical and paraclinical features, treatments, and outcomes of the five patients in this study. All patients were engaged in dietary weight loss at the time of disease onset. The mean age of the patients was 30.2 ± 12.9 years. The average duration from the onset of neurological symptoms to medical consultation was 14.2 ± 5.2 days, with all cases presenting acute onset (within 4 weeks). Weight loss ranged from 5 to 20 kg, and none of the patients had a history of alcohol consumption, diabetes, peptic ulcer disease, or gastrointestinal surgery.

**Table 1:** Clinical characteristics of the five diet-induced GBS cases.

Data	Case 1	Case 2	Case 3	Case 4	Case 5
Age/gender, years	48/M	26/F	37/F	14/M	26/F
Onset-to-admission (days)	18	13	3	21	9
Preceding infection	Diarrhea	None	Diarrhea	Diarrhea	None
Weight loss (kg)	5	10	10	10	20
Duration of weight loss (days)	10	60	60	30	75
Time to nadir (days)	7	5	3	16	4
Symptoms at nadir	Paraplegia, limb pain, areflexia	Quadriplegia, paresthesia, areflexia	Tetraparesis, sensory loss in all limbs	Paraplegia, paresthesia, areflexia	Paraplegia, limb pain, areflexia
mRS at nadir	1	1	4	2	1
Cranial nerve deficits	None	None	Facial nerve	None	None
Serum albumin (g/L)	45	35	36	37	40
WBC in CSF (cells/μL)	3	2	4	2	3
CSF protein (g/L)	1.67	0.37	0.51	0.11	0.28
Nutrient deficiency	No	No	No	No	No
Nerve conduction study	AMAN	AMSAN	AIDP	AMSAN	AMSAN
Sural nerve sparing	No	No	Yes	No	No
FWL (ms)					
Tibialis (L/R)	53.7/52.1	NP/NP	49.5/51.2	43.4/44.3	48.7/50.9
Median (L/R)	24.6/25.3	-	24.5/25.1	-	24.9/25.4
Ulnar nerve (L/R)	-	20.7/20.5	-	21.8/21.4	-
Antig-anglioside antibodies	Negative	Negative	Not tested	Not tested	Not tested
Treatment	B-complex vitamins, physical therapies	IVIG, physical therapies	IVIG, physical therapies	IVIG, physical therapies	B-complex vitamins, physical therapies
Prognosis	Complete recovery	Complete recovery	Complete recovery	Complete recovery	Complete recovery

mRS, modified Rankin Scale; CSF, cerebrospinal fluid; IVIG, Intravenous Immunoglobulin; AMAN, Acute Motor Axonal Neuropathy; AMSAN, Acute Motor and Sensory Axonal Neuropathy; AIDP, Acute Inflammatory Demyelinating Polyneuropathy; FWL, F wave latency; NP, No potential; WBC, White Blood Cell Count. Nutrient deficiencies include deficiencies in vitamin B1, vitamin B12, folate, calcium, and vitamin D. Anti-ganglioside antibodies include serum anti-GQ1b antibody, anti-GD1b antibody, anti-GM3 antibody, and anti-GM1 antibody (IgG, IgM). The normal range for CSF protein is 0.15–0.45 g/L. The normal CSF WBC count should be less than 5 cells/μL. F-wave latencies normal value: Tibialis < 55 ms; Median < 26 ms.

All patients experienced acute four-limb weakness and altered sensation in either the lower limbs or both upper and lower limbs. Four patients had low albumin levels, indicative of malnutrition, while levels of vitamin B1, vitamin B12, folate, calcium, and vitamin D were normal in all patients. Electrophysiological findings were consistent with GBS, demonstrating pronounced axonal involvement. Cerebrospinal fluid (CSF) analysis revealed albuminocytologic dissociation in two patients. Serum and cerebrospinal fluid tests for metabolic, infectious, and autoimmune causes of acquired peripheral neuropathy were negative. Serum *C. jejuni* antibodies and stool cultures were also negative. Furthermore, all stool cultures for common diarrhea-associated pathogens were negative. The mean time to reach the disease nadir (signifying rapid clinical deterioration) was 7.0 ± 5.2 days. One patient (Case 3) required support for walking at the peak of disease severity, while the other four presented milder symptoms. Cases 2, 3, and 4 received Intravenous Immunoglobulin (IVIG) (2 g/kg over 3–5 days), whereas Cases 1 and 5 received nutritional therapy without IVIG. At the six-month follow-up, all patients achieved full recovery, with no residual neurological deficits reported.

### 3.2 Literature review and comparison with bariatric surgery-induced GBS cases

A systematic literature review identified nineteen cases of GBS following bariatric surgery, summarized in Table 2. These cases primarily involved surgical procedures such as Sleeve Gastrectomy (SG), Laparoscopic Gastric Restriction Surgery (LGRS), Laparoscopic Gastroplasty (LGP), Roux-en-Y Gastric Bypass (RYGB), and Post-Distal Gastrectomy with Gastroduodenal Anastomosis (Post-DG-GDA). The weight loss associated with these cases ranged from 25 kg to 90 kg, with the onset of GBS symptoms occurring between 1 to 12 months post-surgery. Clinical presentations included paraplegia, quadriplegia, paresthesia, areflexia, and in some cases, facial nerve deficits and respiratory failure. Treatments varied among patients, including IVIG, plasma exchange (PE), gabapentin, and nutritional supplementation. Prognoses were generally favorable, with most patients achieving full or nearly full recovery within several months, although some had lingering deficits such as the need for a cane or persistent weakness.

**Table 2:** Summary of GBS following bariatric surgery: clinical features and treatment.

Case	References	Age/sex	Surgical procedure	Surgery to-onset (months)	WL (kg)/duration (mo)	Postop Vit. Suppl. (Y/N)	Prev. Inf./Spec. microbe	Symptoms at nadir	CSF protein (g/L)	CSF WBC (cells/μL)	Anti-Ganglioside Ab	EMG/NCS	Nut. assess.	Treatment	Prognosis
1	Şahin Ş (15)	27/F	SG	3	20/3	Yes	No/NA	Paraplegia, limb pain, areflexia	Normal	Normal	NA	AMSAN	Normal Vit. B12/Folate and metabolic panel	IVIG, B-complex vitamins	Walking short distances
2	Şahin Ş (15)	19/M	SG	4	90/6	Yes	No/NA	Quadriplegia, paresthesia, areflexia	Normal	Normal	NA	AMSAN	Normal Vit. B12 and folate	IVIG, plasmapheresis, B-complex vitamins, physical therapy	Nearly normal
3	Machado FCN (21)	25/M	LGP	1	21/1.5	No	No/NA	Paraplegia, paresthesia, areflexia	Normal	Normal	No	Sensory-motor polyneuropathy	Low Vit. B6 (pyridoxine) and B1 (thiamine) levels	IVIG, B-complex vitamins	Independent ambulation
4	Sunbol AH (22)	20/F	SG	4	30/4	NA	Upper respiratory infection/NA	Bilateral lower limb weakness and numbness	0.31	1	NA	Absence of F waves, axonal involvement	Normal Vit. B12	Intubation, IVIG, vitamin supplementation	Needs cane for walking
5	Sunbol AH (22)	36/F	SG	12	40/2	No	Diarrhea/NA	Quadriplegia, decreased lower limb reflexes	NA	NA	NA	AMAN	Normal Vit. B12 and D	IVIG, vitamin supplementation, physiotherapy	Weakness persists after discharge
6	Sunbol AH (22)	22/F	LSG	1	(Initial BMI 43, final BMI 34.6)/NA	NA	No/NA	Bilateral lower limb paralysis and sensory impairment	0.8	3	NA	Axonal involvement	Vit. B12, Copper, Selenium: Normal Vit. D, Vit. B1: Low K: 2.9 mmol/L Albumin: 31 g/L	IVIG, Nutritional support	Full recovery
7	Ishaque N (19)	30/F	SG	1.5	25% EBW/NA	Yes	No/NA	Quadriplegia, absent reflexes	NA	NA	NA	GBS (demyelinating type)	Normal Ca, P, Vit. B12	IVIG	Complete recovery
8	Aluka KJ (34)	40/F	RYGB	5	Not reported	NA	No/NA	Mild bilateral numbness, 3/5 strength, decreased sensation, areflexia	Normal	Normal	NA	Axonal GBS	Normal folate, Vit. B12; hypoproteinemia	Gabapentin, physical rehabilitation	Full recovery
9	Aljthalin (14)	21/M	Post-DG-GDA	4	47/4	NA	No/No	Bilateral lower limb weakness, feet numbness and burning, balance difficulties	Mildly elevated	Normal	No	AMAN	Normal Vit. B12 and metabolites (MMA, homocysteine), B1, D and folate	IVIG, rehabilitation	Walks with a cane, maintains balance
10	Aljthalin (14)	23/F	LSG	8	54/8	NA	Diarrhea/No	Dysphagia, bilateral lower limb numbness, respiratory failure	Normal	Normal	No	AMSAN	Normal Vit. B12 and metabolites (MMA, homocysteine), vitamins B, D, B6 and folate	IVIG, intubation, rehabilitation	Able to raise limbs against gravity
11	Shehabeldin M (36)	45/F	SG	1.5	NA/1.5	NA	No/NA	Inattention, disorientation, staring spells, areflexia, decreased muscle tone	Elevated	Normal	NA	Axonal GBS	Normal Vit. B12 and metabolites (MMA, homocysteine), vitamins B, D, B6 and folate	IVIG, thiamine	Improvement
12	Chang CG (37)	33/F	OOSGB	3	23/2	NA	NA/NA	Severe limb weakness and hyporeflexia	0.86	Normal	NA	Axonal polyneuropathy with slowed NCV without significant demyelination	Albumin 3.2 g/dL (normal 3.9–4.9); normal Vit. B12 and folate	Plasmapheresis IVIG	Partial lower limb recovery; wheelchair-dependent; persistent upper limb weakness and tremor
13	Chang CG (37)	43/F	OVSGB	3	32/3	NA	No/NA	Upper limb strength 4/5 (weaker distally), lower limb strength 2/5; diminished light touch, pinprick and vibration below elbows; absent DTRs	NA	NA	NA	Severe acute diffuse denervation	Normal Vit. B12; low albumin (2.6 g/dL)	IVIG, rehabilitation	Ambulation restored to baseline after 8 weeks rehabilitation
14	Najjari K (38)	32/F	VBG + GGA	12	NA/12	NA	NA/NA	Obvious tetraparesis, absent deep tendon reflexes	NA	NA	NA	AIDP		IVIG, thiamine, Vit. B6, B12, selenium, etc.	Full recovery
15	Hamdeh MA (39)	20/F	LSG	3	NA/3	NA	NA/NA	Ascending bilateral upper and lower limb weakness and numbness, with blurred vision	0.16	0	NA	Reduced amplitude in left fibular (EDB) CMAP; absent F wave	No abnormalities	IVIG, B-complex vitamins, trace elements	Marked improvement
16	Hamdeh MA (39)	28/F	LSG	3	NA/3	NA	NA/NA	Gradual bilateral lower limb weakness and numbness extending to the umbilical area	NA	NA	NA	NA	NA	Multivitamins, physiotherapy	Able to resume ambulation; slow recovery
17	Ates MP (33)	21/F	LSG	1	NA/3	NA	NA/NA	Upper limb strength 3/5 bilaterally, lower limb strength 2/5 bilaterally, absent DTRs	0.4	Normal	NA	Reduced CMAP amplitudes in fibular and posterior tibial nerves; motor axonal neuropathy	Normal Vit. B12, Vit. E, folate, selenium and copper (pre-treatment initiated)	IVIG, vitamin replacement	Partial improvement
18	Mahwish N (40)	27/F	LSG	2	NA/2	No	No/NA	Distal upper limb strength 4/5, distal lower limb strength 3/5 with distal sensory deficits	Normal	Normal	No	Axonal GBS	Mg 0.91 mg/dL (normal 1.7–2.3), normal Vit. D and Vit. B12	IVIG, physical rehabilitation	Independent ambulation
19	Landais (41)	24/F	Gastric bypass	5	42/5	Poor compliance	NA/*C. jejuni* (residual)	Major tetraparesis, pain in heels and calves	0.36	1	Positive for anti-GM1, anti-MAG, anti-GD1a	AMAN	Vit. B6 and folate deficiency	IVIG, rehabilitation therapy	Partial recovery, can walk with limping

SG, Sleeve Gastrectomy; LGP, Laparoscopic Gastric Plication; LSG, Laparoscopic Sleeve Gastrectomy; RYGB, Roux-en-Y Gastric Bypass; Post-DG-GDA, Post-Distal Gastrectomy with Gastroduodenal Anastomosis; OOSGB, Open Obesity Surgery Gastric Bypass; OVSGB, Open Vertical Sleeve Gastrectomy; VBG + GGA, Vertical Banded Gastroplasty + Gastrojejunostomy; BMI, Body Mass Index; WL, Weight Loss; mo, months; EBW, Excess Body Weight; Postop Vit. Suppl., Postoperative Vitamin Supplementation; Y/N, Yes/No; Prev. Inf., Preceding Infection; Spec. Microbe, Specific Microbial Infection; CSF, Cerebrospinal Fluid; WBC, White Blood Cells; Anti-Ganglioside Ab, Anti-ganglioside Antibodies; EMG, Electromyography; NCS, Nerve Conduction Study; Nut. Assess., Nutritional Assessment; IVIG, Intravenous Immunoglobulin; PE, Plasmapheresis; GBS, Guillain-Barré Syndrome; AIDP, Acute Inflammatory Demyelinating Polyneuropathy; AMAN, Acute Motor Axonal Neuropathy; AMSAN, Acute Motor and Sensory Axonal Neuropathy; MMA, Methylmalonic Acid; CMAP, Compound Muscle Action Potential; SNAP, Sensory Nerve Action Potential; DTR, Deep Tendon Reflexes; EDB, Extensor Digitorum Brevis; NA, Not Available/Not Applicable.

As shown in Table 3, several distinctions emerge when comparing the clinical characteristics and treatment outcomes of dietary weight loss-induced GBS cases with those of bariatric surgery-related GBS. The diet-induced cases had a higher incidence of preceding infections, a higher rate of hypoalbuminemia, slower weight loss rates, and a lower incidence of nutrient deficiencies. Additionally, diet-induced cases generally involved less severe neurological deficits at nadir, with recovery around 6 months appearing more favorable. In contrast, bariatric surgery-associated cases often presented with more severe symptoms, such as quadriplegia and respiratory failure, and required more intensive treatments like PE and prolonged hospitalizations. Despite these differences, both groups shared similar neurophysiological findings, with most cases being of the axonal-type GBS. CSF protein levels were mostly normal, and most patients received IVIG treatment, with overall good prognoses.

**Table 3:** Comparison of clinical characteristics and treatment outcomes between dietary weight loss and weight loss surgery in patients with GBS.

Weight loss method	Dietary weight loss (5 cases)	Bariatric surgery (19 cases)
Preceding infection (*n*, %)	3 (60%)	3 (15.8%)
Weight loss rate (kg/day)	0.17–0.5	0.22–0.7
Percentage of axonal-type GBS (%)	80%	78.9%
Hypoalbuminemia (%)	80%	21.1%
Nutrient deficiency (%)	0	21.1%
Positive ganglioside antibody (*n*)	0	1
IVIG (*n*, %)	3 (60%)	17 (89.5%)
Comparison of IVIG and nutritional supplementation prognosis	No significant difference	Cannot be compared

Axonal-type GBS refers to a form of GBS primarily characterized by axonal damage rather than demyelination. This includes conditions such as Acute Motor Axonal Neuropathy (AMAN) and Acute Motor Sensory Axonal Neuropathy (AMSAN), where the primary pathology involves injury to the axonal structures of peripheral nerves.

## 4 Discussion

This study reviewed five cases of GBS associated with diet-induced weight loss and compared them with nineteen cases of bariatric surgery-related GBS reported in the literature. All patients exhibited acute, symmetrical limb weakness and diminished tendon reflexes.

### 4.1 Trigger mechanisms: preceding infections or weight loss?

In patients with diet-related GBS, 60% reported a preceding infection, while only 15.8% of those with bariatric surgery-related GBS had a similar history. However, despite this difference, it is important to note that all preceding infections in our study were cases of diarrhea, and both serological tests for *Campylobacter jejuni* antibodies and stool cultures were negative. Through stool culture, common diarrhea-associated pathogens were consistently not detected, suggesting that infection may not be the primary trigger in these cases of GBS. We acknowledge that diarrhea could be caused by other pathogens, and the detection of such pathogens may be limited by the range of tests used or variations in pathogen load. Nonetheless, the negative microbiological results do not entirely exclude an infectious trigger due to current diagnostic limitations.

Given these limitations in pathogen detection, alternative triggers must be considered. Furthermore, in the 19 cases of GBS related to weight loss surgery, there is insufficient direct evidence to suggest that a preceding infection triggered the onset of GBS. Among these, two patients had a history of preceding diarrhea, and one had an upper respiratory infection. However, no specific pathogen was identified, and only one case showed a low concentration of *Campylobacter jejuni* antibodies in the serum, with no history of preceding infection.

Moreover, our five diet-induced cases exhibited weight loss rates ranging from 0.17 to 0.5 kg/day, while the literature reports weight loss rates of 0.22 to 0.75 kg/day in bariatric surgery-related GBS cases—far exceeding the recommended safe weight loss rate of 0.5–1 kg per week (24, 25).

Although current evidence is limited, clinical experience suggests that the rapidity and method of weight loss, such as extreme restrictive diets, may predispose patients to nutritional deficiencies and nerve damage. Therefore, given this data, it is plausible that weight loss—whether through dieting or surgery—may itself act as a potential trigger for GBS, independent of infection.

### 4.2 Is malnutrition related to axonal injury?

In patients with diet-related GBS, 80% showed hypoalbuminemia, while only 21.1% of surgery-related GBS patients experienced hypoalbuminemia, and the same percentage had nutritional deficiencies. Despite this, both groups exhibited around 80% of cases with pronounced axonal involvement, and the majority maintained normal CSF protein levels, which is characteristic of axonal forms of GBS. This suggests a potential link between malnutrition and axonal damage in GBS. These findings are consistent with previous reports of peripheral neuropathy following bariatric surgery, where the underlying pathogenesis may differ from classical GBS (26).

Earlier studies have found that acute axonal neuropathy in weight-loss patients is often caused by nutritional deficiencies (27). Malnutrition and acute axonal neuropathy are closely associated, with improvements observed following weight gain and vitamin supplementation. Specifically, deficiencies in iron, calcium, vitamin B12, and fat-soluble vitamins (A, D, E, K) are thought to impair neural function directly, further supporting the idea that malnutrition plays a significant role in axonal injury (28, 29).

### 4.3 The impact of malnutrition on the immune system

Malnutrition significantly impairs the immune defense mechanisms of the host, particularly in the gut epithelium. Nutritional deficiencies restrict the availability of complement components, thereby affecting the capacity of professional phagocytes to engulf and eliminate pathogens. In experimental protein-energy malnutrition (PEM) models in mice, macrophage phagocytosis, as well as the production of reactive oxygen intermediates (ROIs) and reactive nitrogen intermediates (RNIs), is diminished. Additionally, the antigen-presenting function of dendritic cells to T cells is suppressed. In experimental peritonitis in mice, short-term PEM leads to impaired immune cell migration and extravasation, as evidenced by a reduced number of CD11b/CD18-positive cells at the infection site. This impairment may be linked to a decrease in the chemokine MIP-2 concentration (42).

Both innate and adaptive immunity are compromised by malnutrition. Defects in innate immunity include impaired epithelial barrier function of the skin and gut, reduced granulocyte microbicidal activity, fewer circulating dendritic cells, and decreased complement proteins. However, leukocyte numbers and acute-phase responses remain intact. On the other hand, defects in adaptive immunity are characterized by reduced soluble IgA levels in saliva and tears, atrophy of lymphoid organs, diminished delayed-type hypersensitivity responses, a decrease in circulating B cells, a shift from Th1-associated cytokines to Th2-associated cytokines, and lymphocyte hyporesponsiveness to phytohemagglutinin. Nonetheless, lymphocyte and immunoglobulin levels in peripheral blood are preserved (30). Vitamin D deficiency is commonly observed in patients with GBS, and its immunoregulatory role, particularly in the modulation of T lymphocyte function, has been widely recognized (31).

### 4.4 Is IVIG treatment absolutely necessary for diet-induced GBS?

We report five cases of diet-induced GBS, three of which were treated with IVIG and demonstrated favorable outcomes. The remaining two patients, with milder symptoms, received nutritional supplementation (Vitamin B1, B12, and folic acid) and achieved full recovery, despite normal serum levels of these vitamins. These findings suggest that IVIG treatment may not be absolutely necessary in diet-induced GBS patients. Similar to axonal peripheral neuropathy seen after bariatric surgery, favorable outcomes can often be achieved through nutritional supplementation alone (27, 32).

Currently, there are no guidelines or comparative studies supporting the necessity of IVIG treatment for diet-induced GBS. However, in bariatric surgery-related GBS, one case showed a positive anti-ganglioside antibody result (41), suggesting that an immune mechanism may be involved in the pathogenesis of GBS and supporting the rationale for IVIG treatment. Given that GBS can be life-threatening and lead to permanent disability, a combined treatment approach of IVIG and nutritional supplementation might be a prudent choice, depending on the severity of the symptoms, the rate of progression, and the patient’s preferences. Further large-scale comparative studies are needed to explore the necessity of IVIG treatment in diet-induced GBS.

## 5 Conclusion

Both diet-induced weight loss and bariatric surgery may trigger GBS. It is essential for clinicians to recognize the early signs of GBS in patients undergoing these weight loss interventions and provide prompt treatment to improve outcomes and prevent complications.

### 5.1 Limitation

The primary limitations of this study include the small sample size and its retrospective, single-center design, which may introduce selection bias and incomplete data issues. It is important to note that our hospital is part of a larger regional network—including major institutions such as Xiangya Hospital—that treats a significantly higher number of GBS cases; thus, calculating incidence solely from our hospital’s data may not fully reflect the regional burden. Future studies should incorporate epidemiological expertise and involve multi-center data to better ascertain the relationship between rapid weight loss and GBS. Therefore, conclusions should be interpreted with caution and validated through larger-scale prospective studies.

## Data Availability

The datasets presented in this article are not readily available because of ethical and privacy restrictions. Requests to access the datasets should be directed to the corresponding author.
